# Evaluation of Project P.A.T.H.S. in Hong Kong: Utilization of Student Weekly Diary

**DOI:** 10.1100/tsw.2008.2

**Published:** 2008-01-14

**Authors:** Daniel T. L. Shek, Rachel C. F. Sun, Ching Man Lam, Daniel W. M. Lung, Sui Chung Lo

**Affiliations:** ^1^ Centre for Quality of Life, Hong Kong Institute of Asia-Pacific Studies, The Chinese University of Hong Kong, Hong Kong; ^2^ Kiang Wu Nursing College of Macau, Macau; ^3^ Social Welfare Practice and Research Centre, The Chinese University of Hong Kong, Hong Kong

**Keywords:** adolescence, youth development, Chinese, Hong Kong

## Abstract

Four schools participating in the experimental implementation phase of the Project P.A.T.H.S. (Positive Adolescent Training through Holistic Social Programmes) (Secondary 1 level) were randomly selected and invited to join this research study. After completion of the Tier 1 Program, Secondary 1 students in the participating schools were invited to write a reflective journal in the form of a weekly diary in order to reveal their perceptions and feelings regarding the Tier 1 Program and the related benefits. Results of the qualitative data analyses showed that most of the respondents (a) had positive views on the program, (b) had positive views on the instructors, and (c) stated that they had acquired competencies at societal, familial, interpersonal, and personal levels after joining the program. The present qualitative findings based on students' weekly diaries provide additional support for the effectiveness of the Tier 1 Program of P.A.T.H.S. in Hong Kong.

## INTRODUCTION

Anne Frank's diary is a famous book that records the events, thoughts, feelings, and dreams of a Jewish teenage girl who had fled and hid in Amsterdam with her family when the Germans conquered the Netherlands. This diary is popular because it is a document that reflects an adolescent's development and, at the same time, reveals the historical events of the Holocaust during World War II. In view of the invaluable information gathered from people's diaries, dairies for analyzing human behavior, discovering history, or evaluating a program represent an attractive research approach.

In the context of social sciences research, a diary is commonly used in nonexperimental research to collect data on human behavior. For example, Leigh[1] asked participants to record the time, quantity, and type of drinking and sexual activities over 10 weeks in order to examine the temporal relationships of alcohol consumption to sexual behavior. Behavioral diaries have also been used in behavioral modification programs[2]. There is research that suggests the strengths and weaknesses of using daily diaries as a research strategy[3,4] and the debate has continued in the literature[5,6].

In the field of education, teachers are generally encouraged to be reflective practitioners, and they are expected to keep a diary or reflective journal that records their lesson planning and reflection on the teaching processes. Hence, it is not uncommon to find that a teacher would keep reflective journals as a means to assess personal teaching performance, identify one's pedagogical strengths and weaknesses, and evaluate the effectiveness of the changes implemented in teaching[7]. It is believed that a reflective journal "suggests questions, identifies new areas to explore, reveals meaningful absences, and uncovers recurring patterns" [8 (p. 16)]. Therefore, some studies utilized teachers' reflective diaries as a kind of data collection method in order to investigate the essential components of effective teaching practice[9] or pedagogy[10], and to examine ways in which to contribute to professional development[11].

Similarly, in contrast to the traditional learning model, where students mainly take a passive role in receiving what their teachers transmitted or reciting the information in books, students are perceived as active learners who construct their personal knowledge based on their own experiences in reflective learning models, where they are encouraged to be autonomous learners who reflect on their learning and to have more awareness of their learning processes, competencies, and limitations. Therefore, reflective observation is highlighted to be an indispensable process to translate concrete experiences into concepts and action in the experiential learning process[12], in which keeping a diary is considered as an opportunity for personal reflection that, in turn, enhances the students' autonomy and develops an ownership of their learning process[13].

In order to guide students to become autonomous learners, students participating in human services programs are commonly asked to write diaries or reflective journals in different ways. First, some courses simply aim to encourage students to be critical and reflective learners. For example, in a module on management education, students were asked to write eight diaries with predesigned topics throughout the course[14]. Second, some courses aim to strengthen students' ability to integrate their course learning into the actual field settings. For example, social work students were required to write reflective journals with predesigned questions at several stages of their field work, on top of other written assignments[15]. Third, students can be guided to be masters of their future. For example, students in a health discipline course were asked to write a journal with no specific guidelines on their experiences of their practices in rural areas so as to identify the positive and negative aspects of rural practices, and develop their interest in working in the rural areas in the future[16]. Chaloner[17] affirmed that "using a personal journal implies that the learner will construct a memory of the learning experience and how it can be applied to the workplace by using a personal narrative. Through such an individualized and personal approach, it is hoped that the learner will be motivated enough to assume the autonomy that he/she is being offered" (p. 22).

In addition, there are studies that use diaries or reflective journals as an evaluation strategy. In some studies, terminal reflective journals were often used as one of the information sources to evaluate the effectiveness of a program in order to suggest improvement in the planning and management of similar programs in the future[18,19,20]. On the other hand, periodic reflective journals were commonly used in evaluation, where students who kept writing journals were also found to have deeper understandings and could apply concepts to their daily lives[21,22]. For this reason, writing periodic reflective journals was regarded as a promising process evaluation or subjective outcome evaluation strategy[23].

In Hong Kong, there are very few research studies that document the use of diaries or reflective journals as a form of data collection, particularly in the education field[24,25]. Interestingly, in most of the primary and secondary schools in Hong Kong, students are commonly required to write weekly diaries and submit them to their class teachers, who will then write down their comments after reading the weekly diaries. With the ease of collecting students' weekly diaries in the secondary schools in Hong Kong, the present study utilized students' weekly diaries as a research strategy to evaluate the effectiveness of the Project P.A.T.H.S. (Positive Adolescent Training through Holistic Social Programmes). Several schools that joined P.A.T.H.S. were invited to ask their students to write down their perceptions and feelings after joining the Tier 1 Program in one of the weekly dairies. As the project had already collected students' views on the program in terms of quantitative data generated from structured questionnaires, as well as qualitative data generated from the open-ended questions from questionnaires and focus group interviews, it is hoped that the information collected from weekly diaries can generate further qualitative data for understanding the students' perceptions of the program in a comprehensive manner.

## METHODS

### Participants and Procedures

In 2005/2006, 52 schools joined the Tier 1 Program (Secondary 1 level) of the experimental implementation phase of P.A.T.H.S. In the Tier 1 Program, 29 schools adopted the full program (i.e., 20-h program involving 40 units) and 23 schools adopted the core program (i.e., 10-h program involving 20 units). After completion of the Tier 1 Program, four schools were randomly selected and invited to join this research study. The teachers were asked to invite randomly some Secondary 1 students to write a journal in the form of a weekly diary in order to reveal their perceptions and feelings after joining the Tier 1 Program, or to select randomly the submitted weekly diaries to the research team. Although the students' identities were revealed when they handed in the weekly diaries to their teachers, their identities remained anonymous when a copy of their work was sent to the research team. The students were informed of the purpose of this study and reassured that the information they had given would be kept confidential and for research use only. The students' willingness to join the study was respected and their consent of participation was sought.

Among the four schools, three adopted the full program, while one adopted the core program. All were coeducational schools. The mean number of Secondary 1 students in these schools was 158.25 (range: 94–240), and each school had four to six classes. The mean number of teachers and social workers who taught the Tier 1 Program per school were 4.23 (range: 3–5) and 2.25 (range: 1–4), respectively. The total number of students' weekly diaries received was 95, which was about 15% of the total Secondary 1 students in these four schools. As the number of cases in qualitative studies is commonly not high, the number of weekly diaries can be regarded as acceptable.

### Instruments

The students were asked to write a journal in the form of a weekly diary in order to reveal their perceptions and feelings after joining the Tier 1 Program. No specific guidelines on how to write and what to write were given. It was homework, and students completed it at home.

### Data Analyses

The data were analyzed using general qualitative analyses techniques[26] by the fourth and fifth authors, and counterchecked by the first and second authors. There were three steps in the data analysis process. First, relevant raw codes were developed for words, phrases, and/or sentences that formed meaningful units at the raw responses level. Second, the codes were further combined to reflect higher-order attributes at the category of codes level. Third, the categories of codes were further analyzed to reveal the broader themes at the thematic level. For example, the response of "the program content is practical" at the raw response level could be subsumed under the category of "program content", which could be further subsumed under the broad theme of "views on program" (see Table 1).

**TABLE 1**
**Views on Program and Instructors**

| Area | Subcategory | Item | N | N in Subcategory | Total N |
|---|---|---|---|---|---|
| Views on program | Overall impression | Perfect/excellent/very good | 8 | 29 | 124 |
| | | Like the program | 7 | | |
| | | The program is good | 3 | | |
| | | The project is meaningful | 2 | | |
| | | Matches adolescents' needs | 2 | | |
| | | Appreciate the program/activities/format | 3 | | |
| | | Better than other programs | 1 | | |
| | | Mixed blessing | 1 | | |
| | | Not different from other programs | 1 | | |
| | | Similar to the subjects in primary schools | 1 | | |
| | Program content | Program content is practical | 13 | 33 | |
| | | Program content can be easily understood | 8 | | |
| | | Diverse topics/rich content | 5 | | |
| | | Creative | 1 | | |
| | | Dislike growth puzzles (negative) | 3 | | |
| | | Insufficient information (negative) | 1 | | |
| | | Too few activities (negative) | 1 | | |
| | | Too many worksheets (negative) | 1 | | |
| | Learning process | Interesting | 8 | 39 | |
| | | Happy | 6 | | |
| | | Can stimulate students' creative responses | 2 | | |
| | | Funny | 6 | | |
| | | Students' active participation | 4 | | |
| | | Relaxed | 7 | | |
| | | Not dull | 1 | | |
| | | Boring (negative) | 5 | | |
| | Other comments and suggestions | Suggestions | 13 | 23 | |
| | | Others | 10 | | |
| Views on instructors | Overall impression | Instructor was pleasing | 2 | 5 | 22 |
| | | Instructor was disgusting (negative) | 3 | | |
| | Teaching performance | Instructor's performance was excellent | 3 | 5 | |
| | | Instructor was well prepared for the program | 1 | | |
| | | Professional teaching | 1 | | |
| | Teaching attitude | Enthusiastic | 1 | 5 | |
| | | Involved | 3 | | |
| | | Willing/able to help students | 1 | | |
| | Teaching process | Teaching was interesting and vivid | 3 | 4 | |
| | | Teaching was clear | 1 | | |
| | Others | Others | 3 | 3 | |
| **TOTAL** | | | | | **146** |

*Note:* (negative) = negative responses (N = 14)

Twenty coded raw descriptors were randomly selected from examining intra- and inter-rater reliability. The raters were required to code the randomly selected descriptors into two categories (i.e., views on program and views on instructors) without knowing the original codes given.

In the present qualitative analyses, because the researchers designed the program in P.A.T.H.S., they were conscious of their own biases and expectation of the program to be effective. As such, the first, second, and third authors (researchers of the study) were not directly involved in the first few steps of the data analyses. In addition, in order to minimize the possible biases involved, both intra- and inter-rater reliabilities on the coding were calculated. For intrarater reliability, the second and fourth authors individually coded 20 randomly selected responses for each question. For inter-rater reliability, a doctoral student and a research assistant with a Master's degree coded 20 randomly selected responses for each question without knowing the original codes given at the end of the scoring process with reference to the codes finalized by the first author.

Following the principles of qualitative analyses[27], the following attributes of the study regarding data collection and analyses are highlighted: (1) a general qualitative orientation is adopted, (2) recruitment process for the participants and justifications for the number of participants are described, (3) details of the data collection are given, (4) the issues of biases and ideological preoccupation are addressed, (5) inter- and intrarater reliabilities information is presented, and (6) the categorized data are kept in by a systematic filing system in order to ensure that the findings are auditable.

## RESULTS

As shown in Table 1, 146 meaningful units regarding the students' perceptions of the Tier 1 Program and the instructors could be categorized into two categories (i.e., views on program and views on instructors). In the aspect of "views on program", there were 124 responses that could be categorized into "overall impression", "program content", "learning process", and "other comments and suggestions". Most of the respondents had positive views on the "learning process" (N = 34), such as "interesting" and "relaxed", while some perceived the learning process as "boring" (N = 5). Many respondents showed their views on the "program content" with many positive comments (N = 27), such as "the program content is practical", and some negative views (N = 6), such as "dislike growth puzzles". On the other hand, there were 22 responses of "views on instructors" that were categorized into "overall impression" (N = 5, including 3 positive comments of "the instructor was pleasing" and 2 negative comments of "the instructor was disgusting"), "teaching performance" (N = 5, e.g., "instructor's performance was excellent"), "teaching attitude" (N = 5, e.g., "involved"), "teaching process" (N = 4, e.g., "teaching was interesting and vivid"), and "others" (N = 3). In total, there were 90.4% positive responses regarding the students' perceptions of the program and instructors. The intrarater agreement percentage was 100% and inter-rater agreement percentage was 85%.

The perceived benefits of the program to the students are shown in Table 2. There were a total of 203 meaningful units that could be categorized in five categories (i.e., societal, familial, interpersonal, personal levels of competence, and others). Most of the respondents reported that they learned personal competence (N = 86), particularly in the subcategory of "positive self-image" (N = 32, e.g., "self-understanding"), following by "moral competence and virtues" (N = 14, e.g., "to distinguish between right and wrong"), "cognitive competence" (N = 12, e.g., "problem-solving"), and "emotional competence" (N = 11, e.g., "emotional management"). Next, many respondents reported that they acquired interpersonal competence (N = 48), which could be categorized into "general interpersonal competence" (N = 42, e.g., "get along with others") and "specific interpersonal competence" (N = 6, e.g., "respect"). The intrarater agreement percentage was 100% and inter-rater agreement percentage was 100%. Below are three case illustrations on the perceived benefits of the program:

- Student A in School 1: "Project P.A.T.H.S. has taught me a lot of things that can be applied in daily life. It enabled me to learn team spirit and co-operation among teammates, as well as to work independently. Every topic could be understood easily. Although my class was noisy, the instructors were passionate in teaching. They would wait until we kept quiet, and this has helped me to learn self-discipline."

**TABLE 2**
**Perceived Benefits to Students**

| Level of Competence | Subcategory | Item | N | N in Subcategory | Total N |
|---|---|---|---|---|---|
| Societal level | Social norms | Following social norms | 3 | 3 | 14 |
| | Social responsibility and affairs | Knowledge about China and Hong Kong | 6 | 11 | |
| | | Citizen's responsibilities | 4 | | |
| | | Knowledge about public affairs | 1 | | |
| Familial level | Family relationships | Get along with family members | 5 | 5 | 5 |
| Interpersonal level | General interpersonal competence | Promoting team spirit | 9 | 42 | 48 |
| | | Get along with others | 19 | | |
| | | Making friends | 7 | | |
| | | To differentiate between healthy and unhealthy friends | 7 | | |
| | Specific interpersonal competence | Caring for and helping others | 1 | 6 | |
| | | Apology | 1 | | |
| | | Politeness | 2 | | |
| | | Respect | 2 | | |
| Personal level | Cherishing life | To cherish life | 1 | 2 | 86 |
| | | To treasure people and things | 1 | | |
| | Reflection | Self-reflection | 3 | 8 | |
| | | Wisdom of life | 5 | | |
| | Cognitive competence | Rational thinking and analytical thinking | 5 | 12 | |
| | | Problem solving | 7 | | |
| | Ways to face adversity | Optimism | 1 | 1 | |
| | Positive self-image | Personal growth | 11 | 32 | |
| | | Self-confidence | 3 | | |
| | | Self-understanding | 18 | | |
| | Emotional competence | Emotional management | 11 | 11 | |
| | Goal setting | Goal setting | 6 | 6 | |
| | Moral competence and virtues | Personal ethics | 2 | 14 | |
| | | Equality and fairness | 3 | | |
| | | To distinguish between right and wrong | 5 | | |
| | | To correct bad habits/defects | 4 | | |
| Others | | Others | 10 | 50 | 50 |
| | | Interest in learning | 1 | | |
| | | Fruitful | 10 | | |
| | | Stimulating | 2 | | |
| | | Learned a lot of knowledge | 14 | | |
| | | Learned something useful | 13 | | |
| **TOTAL** | | | | | **203** |

*Note:* Twenty coded raw descriptors were randomly selected from examining intra- and inter-rater reliability. The raters were required to code the randomly selected descriptors into four categories (i.e., societal, familial, interpersonal, and personal) without knowing the original codes given.

- Student B in School 2: "I think this program has taught me self-cultivation, getting along with friends and attitudes in handling daily matters. I also learnt that problems can be solved when we get united."
- Student C in School 3: "I think Project P.A.T.H.S. was a bit boring, but rather meaningful. It has helped me to understand what is civism, how to control my emotions, prevent conflicts, recognize my talents, set goals and make friends with the healthy peers, etc. This program has enriched my knowledge and cultivated me to control my temper. All in all, this program was good."

## DISCUSSION

Based on the weekly diaries written by the students, the present study showed that the participants generally had positive perceptions of the Tier 1 Program of P.A.T.H.S. and the instructors, and they overwhelmingly regarded the program was beneficial to their own development. The present findings basically concur with both the quantitative and qualitative findings related to subjective outcome evaluation[28,29,30,31], qualitative evaluation findings[32], and process evaluation findings[33,34]. In short, the present findings provide additional support for the effectiveness of the Tier 1 Program of P.A.T.H.S. in Hong Kong. From the perspective of triangulation, the existing evaluation findings suggest that the effectiveness of the Tier 1 Program is supported by evaluation data collected from different sources and by different strategies.

The present findings underscore the utility of using weekly diaries to evaluate positive youth development programs. Actually, there are several strengths of students' diaries and reflective journals. The first strength is that a reflective journal can consolidate the learning experiences of the learners. As pointed out by Goldenhar and Kues[23], "journaling may be an educational intervention itself, because it provides students the opportunities to take time to reflect on their educational experiences" (p.531). In a study conducted by Hsiao et al.[35], after nursing students had learned to practice a healthy life style, they were asked to set up and implement a plan for changing their unhealthy behavior, and to record their behavioral changes daily in a standard format and write reflective journals for 4 weeks. The researchers found that the daily records and reflective journals not only assisted students to become self-monitoring in changing their unhealthy behavior, but also let the nursing students experience the process of behavioral changes so as to develop empathy for their clients and transform the skills in their future careers.

The second strength is that a weekly diary is a naturalistic research method that has high ecological validity[22]. In the study by Schmitz and Wiese[22], it was found that using standardized diaries for students to record their learning goals and to evaluate the strategies used to achieve the goals over 5 weeks could remind students of the process of self-regulation, and most importantly, enabled students to transfer skills learned in schools to home, since the diaries were written in the students' home setting.

The final strength is that reflective journals such as weekly diaries with some guided questions could act as a good tool for educational evaluation, on top of being a learning vehicle for students to reflect on their learning experiences and integrate them into the future careers[23]. Moreover, in addition to the information collected from students' responses for course evaluation, teachers could also be invited to be researchers to record students' changes in several phases of the course in the format of weekly diaries in order to collect multiple data from different data sources[36].

Nevertheless, there are three weaknesses of using weekly diaries. First, although students have the freedom to write down their daily life experiences, feelings, and thoughts in the weekly diaries, it is understandable that some students might have uncomfortable feelings about disclosing their inner world to others and might perceive the diary as an "invasion to privacy"[14]. Second, for students who are not good at using written techniques to express their experiences, this method may not be a good tool. This problem may be particularly acute in schools that admit students with poor academic achievement. This is also a problem in the Chinese culture where Chinese people may lack the language to describe their feelings[37]. In addition, writing weekly diaries requires students' competencies on reflection and critical thinking, but the students may lack experiences in doing reflection. Third, as a qualitative technique, weekly diaries inherit the basic problems of qualitative methods[27].

Although the present findings can be interpreted in terms of the effectiveness of the Tier 1 Program of P.A.T.H.S., there are several possible alternative explanations. First, the findings may not be representative. However, as the schools and participants were randomly selected, this alternative explanation can be dismissed. Second, it is possible that the participants tried to play the role of good participants to please the teachers. Third, the positive findings may be a result of the threats from the teachers and social workers. As we have not checked with the participants, the latter two alternative explanations are plausible. However, as students are normally encouraged to reveal their experience in their submitted weekly diaries and there are no particular reasons for pleasing the instructors, this possibility is not high.

In short, the present study underscores the utility of using weekly diaries to reveal the experiences of participants who join positive youth development programs. In future, some strategies can be employed to improve the quantity and quality of the data collected based on weekly diaries. First, some researchers pointed out that a diary would be a good means of collecting data if clear topics and instructions have been given, or training has been provided for the participants[2,14]. Second, the participants could be helped to master the task better if the length of the weekly diaries could be specified and preferably be lengthened. Finally, instead of asking the participants to give the reflective journal after the completion of the program, it would be more illuminating if the participants could be invited to write periodic reflective journals and/or writing weekly diaries after each unit.

## ACKNOWLEDGMENTS

The preparation for this paper and the Project P.A.T.H.S. were financially supported by the Hong Kong Jockey Club Charities Trust.
